# Gallbladder Rupture in an Adult Weimaraner Dog

**DOI:** 10.1155/crve/6770199

**Published:** 2025-10-20

**Authors:** Armands Vekšins, Ilze Dūzena, Olga Rabočaja

**Affiliations:** Clinical Institute, Faculty of Veterinary Medicine, Latvia University of Life Sciences and Technologies, Jelgava, Latvia

## Abstract

A 6-year-old spayed female Weimaraner weighing 47 kg was referred to the University Veterinary Hospital with suspected acute gastric dilatation and volvulus (GDV). Radiographic examination did not confirm GDV, but a mild peritoneal effusion was diagnosed. Abdominal ultrasound confirmed the diagnosis of peritoneal effusion, but the cause of these changes was not clarified. An increase in leukocytosis and a worsening of the clinical condition were quickly noted. It was decided to perform a CT scan, and localized ileus, gallstones, and the gallbladder showed marked irregular contours and thickening, with suspected rupture as the cause of the peritonitis. A laparotomy confirmed the diagnosis, and surgical treatment was performed.

## 1. Introduction

Peritonitis is an inflammation of the peritoneum, which in most cases has an unfavorable prognosis with a potentially fatal course [1, 2]. Septic peritonitis is predominant [3], and the most common cause of septic peritonitis in dogs is gastrointestinal leakage, but other causes include hepatobiliary disease, intra-abdominal abscesses, uroabdomen, penetrating trauma, and pyometra [4]. Bile peritonitis is a form of this disease in which bile acid leaks from the hepatobiliary system. This disease has been reported in both human and veterinary medicine following rupture of the bile ducts, extrahepatic bile duct obstruction, gallbladder rupture, trauma, or duodenal perforation [5]. The biliary peritonitis diagnostic process is quite difficult and includes diagnostic imaging, such as ultrasound and computed tomography, and laboratory diagnostics [5, 6]. Laboratory diagnostics include biochemical analysis of the abdominal effusion and comparison of the bilirubin concentration with the bilirubin concentration in the blood serum [7].

## 2. Case Presentation

A 6-year-old female Weimaraner weighing 47 kg was referred to the University Veterinary Hospital with suspected acute gastric dilatation and volvulus (GDV). The dog was observed for 2 days with profuse vomiting and excessive salivation. Clumsiness, inability to stand, panting, and loss of appetite were observed. The local veterinarian noted an enlarged abdomen and suspected gastric torsion. It was recommended that the dog be taken to the University Veterinary Hospital.

**Figure 1:**
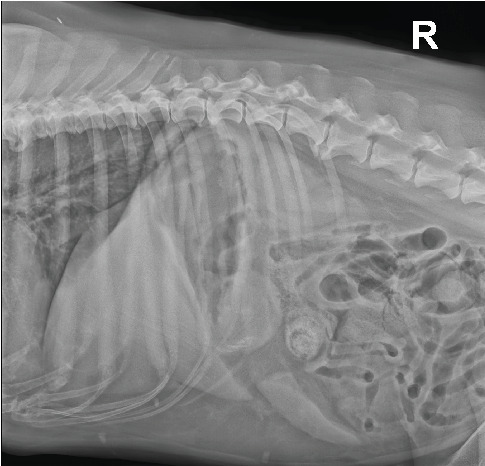
Right lateral abdominal radiograph. Decreased serosal detail is visible in the ventral abdomen, consistent with peritoneal effusion.

**Figure 2:**
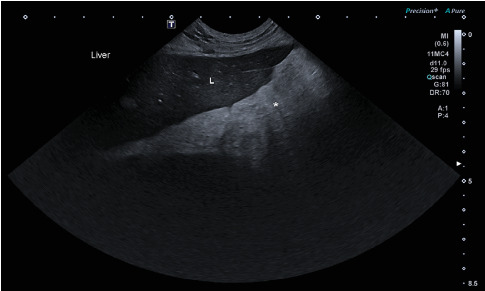
Ultrasound image obtained from the cranial abdomen. Hyperechoic mesenteric fat (∗) is present adjacent to the liver, consistent with regional inflammation.

**Figure 3:**
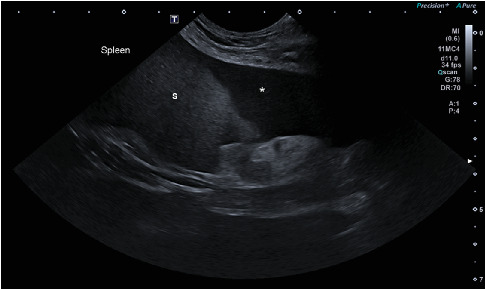
Ultrasound image of the left cranial abdominal quadrant, obtained caudal to the 13th rib. A large volume of peritoneal effusion (∗) with hyperechoic speckles is visible around the spleen (S).

**Figure 4:**
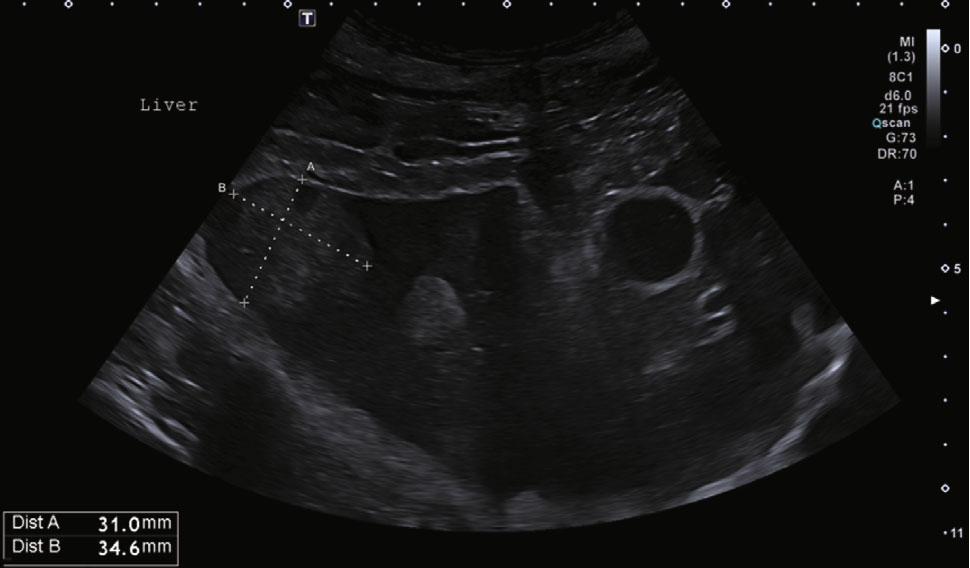
Ultrasound image of the liver. Multiple well-defined, hyperechoic, nodular lesions of varying sizes were present, predominantly within the right liver lobes. The largest lesion measured 31 × 34.6 mm in diameter.

General clinical examination revealed tachypnea (48×/min), moderately elevated body temperature (39.4°C), mild tachycardia (128×/min), and a painful abdomen. Other vital signs were within normal limits. Radiographic examination did not confirm GDV but showed decreased serosal detail in the ventral abdomen, consistent with peritoneal effusion and suspicion of peritonitis (Figure 1). The patient was sent to the ultrasound department where hyperechoic fat (Figure 2) was noted in the cranial abdomen around the liver, spleen, stomach, and kidneys. A large volume of moderately echogenic peritoneal effusion (Figure 3) was seen around the spleen, between the intestinal loops and liver lobes, and around the urinary bladder; multiple well-defined hyperechoic nodular lesions were present throughout the liver (Figure 4)—the lesions varied in size. The largest was 31 × 34.6 mm in diameter. Considering the size of the animal, the wall of the gallbladder was within normal limits (2.7 mm) [8]. A sample of peritoneal fluid was taken. It was reddish, turbid, with 42 g/L total protein, a large amount of degenerative neutrophilic leukocytes, an average number of macrophages, and rare lymphocytes. The result of the laboratory examination was exudate. A biochemical analysis was not performed at this stage. As the ultrasound findings did not clarify the clinical signs and the abdominal effusion, it was decided to perform a CT scan.

**Figure 5:**
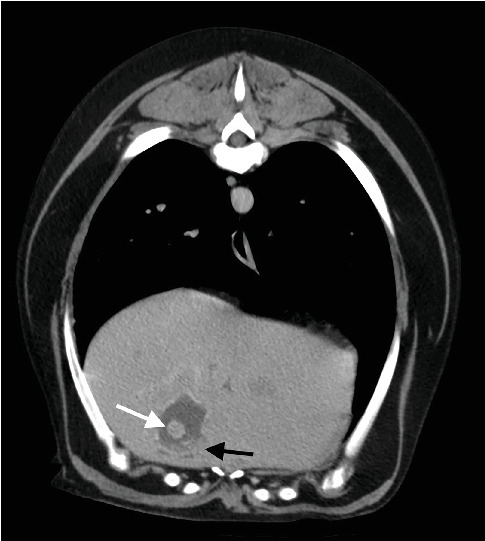
Postcontrast computed tomography image of the abdomen. A hyperattenuating gallstone is visible (white arrow). The gallbladder wall shows irregular contours and thickening (black arrow).

Computed tomography was then performed using a 16-slice MDCT scanner (Philips MX16). The dog was premedicated with butorphanol 0.2 mg/kg intravenously (i.v.) and induced with propofol 4 mg/kg i.v. after 15 min and maintained with inhalation anesthesia (isoflurane). During the examination, the dog was placed in the sternal position, and high-resolution CT scans (120 kV; 259 mA; 2 mm thickness, WL40; WW 350) were performed. Native and postcontrast images (Ultravist, 623 mg/mL [300 mg/mL iodine]) were acquired in the venous and delayed phases. CT findings showed multiple hypodense hepatic nodules, the largest measuring 36 × 28 mm; the gallbladder showed marked irregular contours and wall thickening measuring approximately 4 mm. Diffuse peritonitis was diagnosed based on the presence of peritoneal effusion, fat stranding, and mesenteric enhancement, most pronounced in the cranial abdomen. The hepatic lymph nodes were enlarged (about 15 mm in diameter); the local segment of ileum was 17 mm dilated. The CT findings were pitting cholecystitis; gallstones, based on the presence of hyperattenuating intraluminal structures; suspected rupture of the gallbladder, indicated by irregular contours and focal discontinuity of the gallbladder wall (Figure 5); multiple liver nodules; lymphadenopathy; peritonitis, suggested by mesenteric fat stranding and peritoneal fluid; peritoneal effusion; and a local small bowel ileus. After a CT scan, it was decided to perform a diagnostic laparotomy.

Blood tests (hematology and biochemistry) were performed repeatedly during hospitalization. Hematology showed changes in the number of leukocytes, columnar and segmented neutrophilic leukocytes, and monocytes. Leukocytosis with neutrophilia, a regenerative nuclear shift to the left, moderate toxic changes (including cytoplasmic basophilia, vacuolization, and rare Döhle bodies), and monocytosis were noted before surgery. The deposits were consistent with the inflammatory leukogram.

Biochemical analysis before the operation showed an increase in bilirubin (12.9 μmol/L) and sodium (153.2 mmol/L) and a decrease in total protein (43.36 g/L), albumin (19.21 g/L), and potassium (3.68 mmol/L). It is important to note that the bilirubin concentration in the serum was 6.26 μmol/L 1 day before the operation. The abdominal effusion was biochemically analyzed and compared for bilirubin concentrations with the serum sample. Bilirubin in abdominal effusion was 98 μml/L.

### 2.1. Surgery

A 10-min preoxygenation with 100% oxygen was performed, and the patient was anesthetized according to the standard PIVA protocol. The anesthesia protocol included premedication with acepromazine 0.01 mg/kg i.v. and fentanyl 3 mcg/kg i.v., followed by a CRI of 5–10 mcg/kg/h. Midazolam 0.2 mg/kg i.v. was used as a coinduction agent, followed by induction with propofol 4 mg/kg. After orotracheal intubation, general anesthesia was maintained with isoflurane in 100% oxygen. The positive inotropic drug dopamine 5–10 mcg/kg/min i.v. was administered to maintain mean arterial pressure (MAP) in the normal range. Three milliliters per kilogram per hour of Ringer's lactate solution was infused i.v. throughout the procedure.

**Figure 6:**
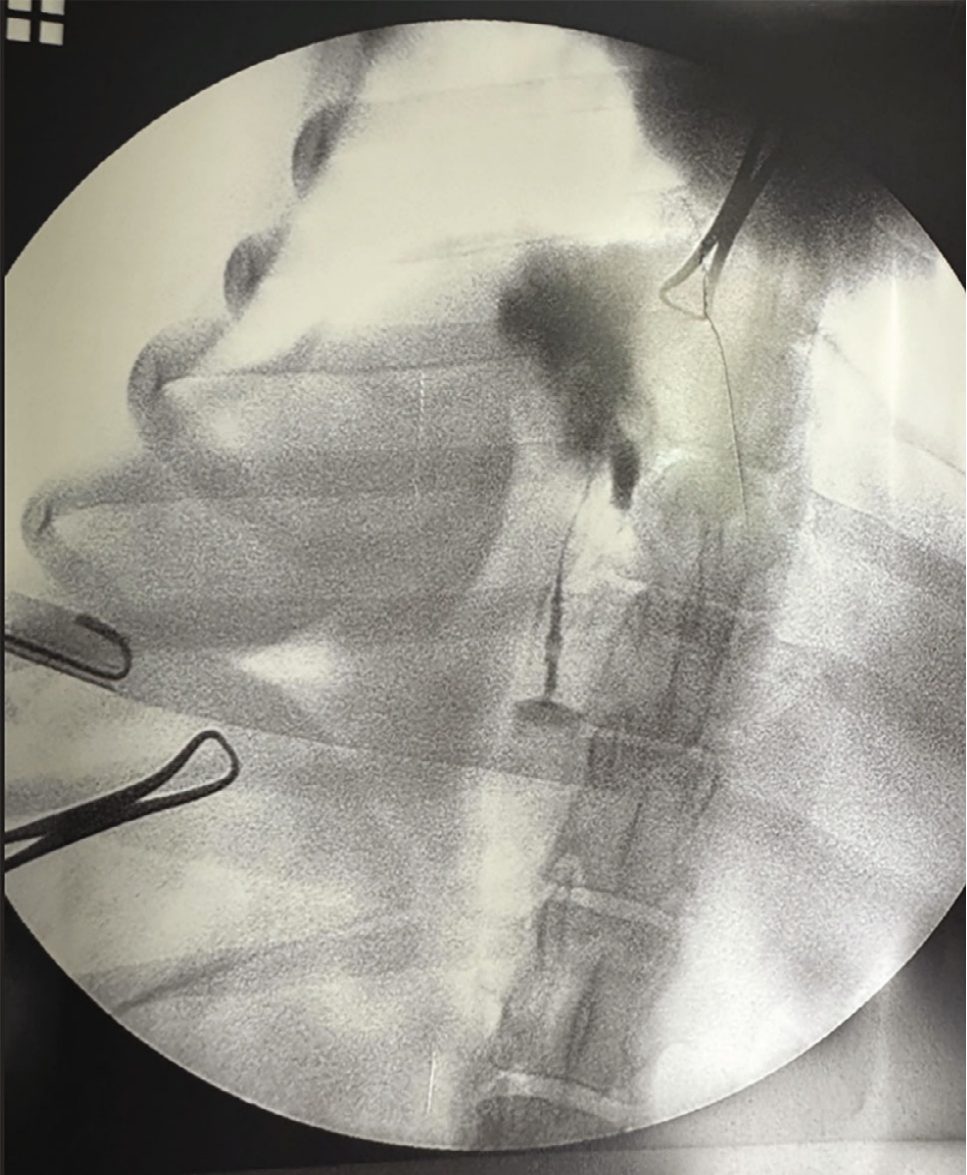
Intraoperative fluoroscopic image following contrast injection into the gallbladder. The site of contrast administration within the gallbladder is visible, with irregular distribution of the medium within the lumen and evidence of extravasation outside the gallbladder wall.

**Figure 7:**
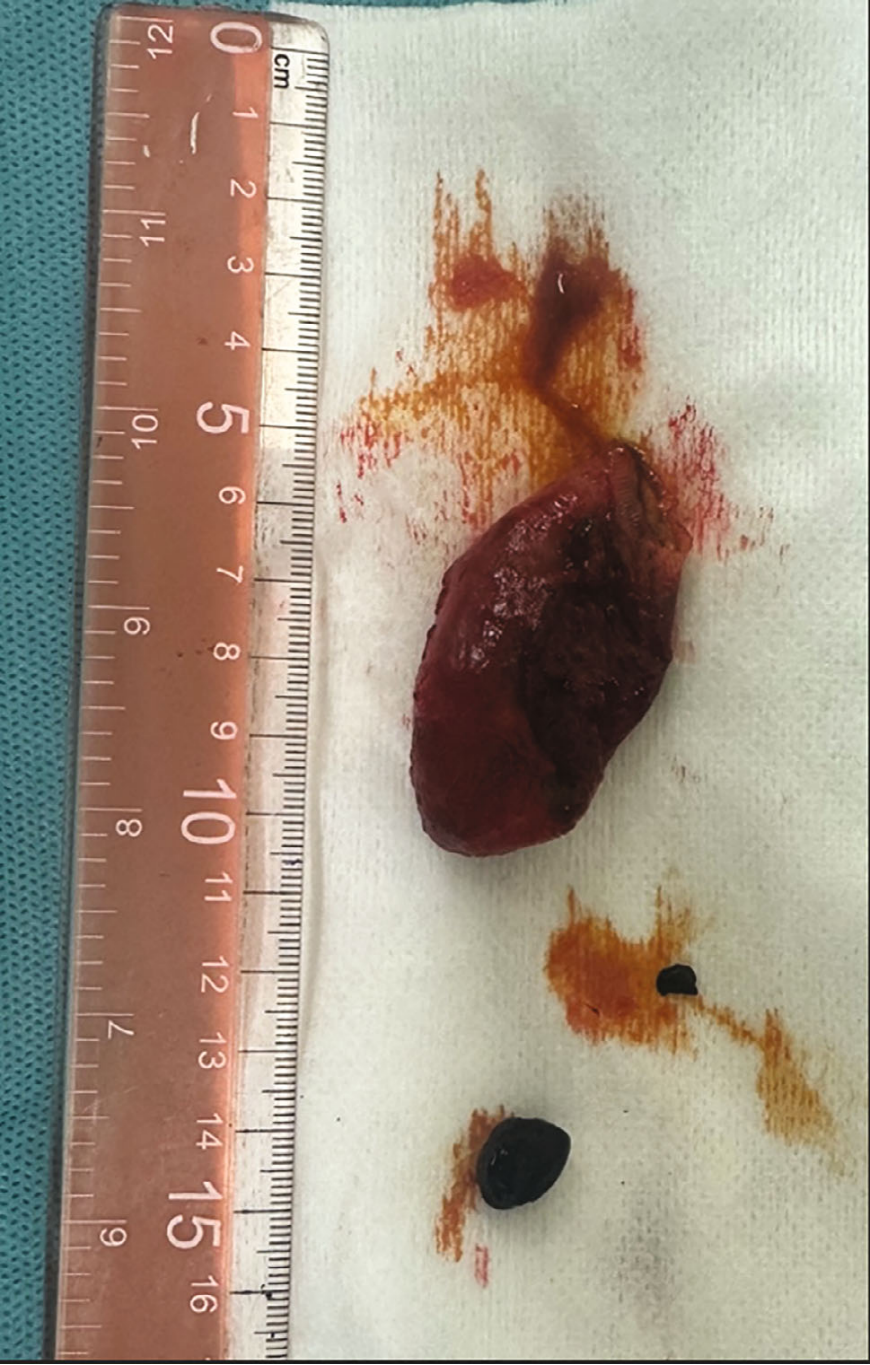
Gross image of the surgically removed gallbladder with choleliths.

The dog was placed in the dorsal position, and the surgical field was prepared for an exploratory laparotomy. The abdomen was opened via a ventral midline incision from the cartilago xyphoidea and distally from the umbilicus. The falciform ligament was removed, and signs of biliary peritonitis were noted. The gallbladder was in the normal anatomical position (between the lobus quadratus and the lobus hepatis dexter). Squeezing the gallbladder did not result in an obvious leakage of bile. The gallbladder was palpated, and bile was found to be covering the surgeon's gloves and coming from the hepatic side of the gallbladder. Because the surgeon wanted to confirm the presence and location of a rupture before proceeding with cholecystectomy, a contrast agent (Urografin 76% [meglumine amidotrizoate/sodium amidotrizoate] mixed 1:1 with NaCl 0.9%, 20 mL volume) was injected into the lumen of the gallbladder. Intraoperative fluoroscopy demonstrated accumulation of contrast medium within the gallbladder and cystic duct, with an irregular gallbladder contour consistent with rupture (Figure 6). Note the irregular shape of the gallbladder. The body of the gallbladder was bluntly separated from the liver, and the laceration (< 5 mm) was obvious. Aspiration of the bile was performed. When the gallbladder was empty, the splint suture was placed, and the dissection of the liver was completed. The neck of the gallbladder was clamped with forceps, and the cystic duct was double ligated with monofilament absorbable suture. After the removal of the gallbladder (Figure 7), bleeding from the liver was stopped with hemostatic gelatin sponges. The sponges were left in the liver for 5 min. Thereafter, no further bleeding was observed. A routine exploratory laparotomy was performed—no other obvious pathology other than small bowel adhesions due to peritonitis was noted. The abdominal cavity was irrigated with copious warm saline, and an open drain was placed prior to closure of the abdominal cavity. The celiotomy was routinely closed with monofilament absorbable sutures. The gallbladder specimen was sent for histopathologic examination, and the findings were necrosuppurative and fibrosing severe cholecystitis with vascular necrosis and thrombosis as well as fibrosuporative peritonitis. The bacteriological results were unremarkable.

### 2.2. Postoperative Care

The dog was hospitalized for a total of 10 days. Antibacterial treatment included amoxicillin/clavulanic acid at 20 mg/kg every 12 h and metronidazole at 10 mg/kg every 12 h. Fluid therapy (RiLac) was administered at 100 mL/h, along with a constant rate infusion of fentanyl-lidocaine-ketamine, which consisted of 2% lidocaine hydrochloride (1 mg/kg/h), ketamine hydrochloride (0.6 mg/kg/h), and fentanyl (4 *μ*g/kg/h). Maropitant citrate was given at a dose of 1 mg/kg every 24 h for the treatment of vomiting and motion sickness.

On the 2nd day after surgery, a blood transfusion was performed because the anemia had progressed (HCT 33.3%; HGB 11.6 g/dL, MCV 64.0 fL, MCH 22.3 pg, MCHC 34.8 g/dL). Prior to transfusion, diphenhydramine 1 mg/kg was administered i.v. Packed erythrocytes 7 mL/kg at a rate of 3 mL per minute were transfused.

On the 3rd day after surgery, the blood test results (HCT 32.9%; HGB 11.3 g/dL, MCV 64.3 fL, MCH 22.2 pg, MCHC 34.5 g/dL) had not improved, and the veterinarian decided to perform a second blood transfusion. After the second blood transfusion, a gradual improvement was observed, and on the day of discharge, the patient's HCT was 35.2%, and HGB was 12.1 g/dL.

After surgery, total protein and albumin levels were monitored. One day after surgery, the total protein was 32.6 g/L, and the albumin was 15.55 g/L. Seven days after surgery, the total protein (43.97 g/L) and albumin (21.8 g/L) were significantly improved. During the treatment period, the dog had severe leukocytosis. Before surgery, the leukocyte count was 54.9 10∗3/ul, and a slight increase (60.1 10∗3/ul) was noted 1 day after surgery. Over the next few days of hospitalization, the total leukocyte count decreased slightly, and on the day of discharge, the leukocyte count was 28.1 10∗3/ul.

### 2.3. Recommendations and Recheck Visit

After discharge, it was recommended to continue the antibacterial treatment for 5 days, and a low-fat diet was prescribed. Blood tests were repeated 10 days after discharge, and the results were unremarkable. On re-examination, the dog was responsive, active, with a good appetite, and without any gastrointestinal tract symptoms.

## 3. Discussion

This case represents a difficult and challenging clinical case in which several diagnostic methods were required for the final diagnosis. Biliary peritonitis is not a common cause of peritoneal inflammation, and identification of a bile acid leak can be challenging. Rupture of the gallbladder is one of the causes of biliary peritonitis [5]. As a gallbladder rupture is a surgical emergency, rapid diagnosis is of crucial importance. The main causes of gallbladder rupture are usually a progressive disease of the gallbladder, for example, a mucocele or cholecystitis; however, abdominal trauma and intraoperative iatrogenic damage can also be a cause of rupture [9]. We have not found many cases in which choleliths are described as the main cause of a gallbladder rupture, as was the case in our instance. However, we suspect that the actual cause is cholelith-caused cholecystitis and thus changes to the gallbladder wall.

The diagnostic process is complicated by the fact that serum bilirubin is not always elevated [10]. In our case, bilirubin levels were initially within the reference range but showed a gradual upward trend, becoming markedly elevated on the day of surgery. Testing bilirubin in the abdominal effusion specimen proved extremely useful, and based on our experience, we recommend performing a biochemical test for peritoneal effusion when the cause is unclear.

Imaging diagnostics play an important role in the diagnosis of abdominal diseases. Ultrasound and computed tomography are the most commonly used methods in small animal practice. In our case, ultrasound examination revealed a large volume of hypoechoic peritoneal fluid with small speckles. These features suggest a cellular effusion, such as a modified transudate, exudate, or fresh hemorrhage, or a chylous effusion. The echogenicity of peritoneal fluid is usually proportional to its content of cells and other debris that act as ultrasound reflectors. Cell-poor fluids, such as transudates, are typically anechoic to hypoechoic compared to exudates, which are typically moderately echogenic [11]. However, there is no way with imaging to diagnose fluid type or cell counts. Therefore, cytology should always be performed on the fluid sample to determine its cellularity and character. During the ultrasound examination, a sample was taken for laboratory examination. The omentum and mesentery appeared thickened, hyperechoic, and hyperattenuating in the cranial abdomen around the liver, spleen, stomach, and kidney. An area of hyperechogenicity of mesenteric fat and localized lymphadenopathy suggests inflammation (steatitis/vasculitis) and should be thoroughly investigated for a possible source of inflammation nearby [11]. This may indicate rupture of the bile ducts with secondary biliary peritonitis, septic peritonitis due to perforation of the gastrointestinal tract, or severe pancreatitis with surrounding peritonitis. These features can significantly limit the penetration of the ultrasound beam and thus the ability to visualize and assess deep organs. Similar limitations may also be observed in cases with a large amount of free fluid. In these cases, the abdominal structures appear ill-defined, and their internal architecture is not well recognized [11]. Several well-defined hyperechoic nodular lesions were visualized throughout the liver parenchyma, which could indicate benign nodular hyperplasia or neoplasia. The lesions varied in size. Considering the size of the animal, the wall of the gallbladder was in the normal range (2.7 mm) or slightly thickened with normal gallbladder volume and anechoic bile. The bile duct could not be visualized due to severe peritonitis. The normal wall thickness of the gallbladder in dogs has been reported to be 1–3 mm [8]. Ultrasound detection of gallbladder wall rupture in dogs with mucocele has a sensitivity of 85.7% and a specificity of 100%. In dogs with more extensive types of gallbladder disease, ultrasound had a sensitivity of 94.4% and a specificity of 44.4%. It appears to be more difficult to detect a rupture in the gallbladder wall without an extruding mucocele or a free mucocele in the peritoneum. Ultrasonographic signs of gallbladder rupture include echogenic fluid around the gallbladder, diffuse echogenic peritoneal fluid, adjacent hyperechogenic fat, inability to confirm continuity of the gallbladder wall, and the presence of a mucocele either protruding from the gallbladder wall or free in the peritoneal cavity [8, 11]. In this case, the ultrasound findings regarding the cause of peritonitis were inconclusive.

In human medicine, multislice computed tomography is considered the best method for diagnosing gallbladder rupture. In dogs with gallbladder rupture, the most typical CT findings are irregular contours of the gallbladder, interruptions in the gallbladder wall, adhesions between the gallbladder and the liver lobes or diaphragm, and diffuse or focal heterogeneous enhancement of the gallbladder wall. Intraluminal gas can also be seen as a CT finding for a gallbladder wall lesion [6].

Ectomy of the gallbladder is a serious procedure. Before it is performed, the veterinarian must be aware of the indications. Despite the CT findings and the radiologist's conclusion that it was a lesion of the gallbladder wall, the surgeon wanted to be sure that the gallbladder had ruptured and so a contrast agent (Urografin 76% [meglumine amidotrizoate/sodium amidotrizoate] mixed 1:1 with NaCl 0.9%, 20 mL volume) was injected into the lumen of the gallbladder to visualize the rupture site. The intraoperative contrast study supported the diagnosis of gallbladder rupture by demonstrating extravasation of contrast medium from the gallbladder wall. In our opinion, this is a useful method to increase diagnostic certainty and surgical safety during cholecystectomy, especially when rupture is not unequivocally evident.

## 4. Conclusion

This case highlights the importance of performing additional diagnostic tests promptly in patients with peritonitis, as every delay in identifying the underlying cause may significantly worsen the prognosis. Abdominal ultrasound is the standard initial examination, but if the result is inconclusive, computed tomography should be considered a valuable option for diagnosing bile peritonitis.

## Data Availability

The data used to support the findings of this study are included in this article.
